# Supplementary material for: Cryo-EM structure of ABCG5/G8 in complex with modulating antibodies
Source: Commun Biol. 2021 May 5;4:526. doi: 10.1038/s42003-021-02039-8 (PMC8100176; doi:10.1038/s42003-021-02039-8)
Supplement: Supplementary file 1 — Supplementary Information [file 42003_2021_2039_MOESM1_ESM.pdf]

Supplementary information

TITLE: CRYO-EM STRUCTURE OF ABCG5/G8 IN COMPLEX WITH MODULATING ANTIBODIES

Zhang *et al.*

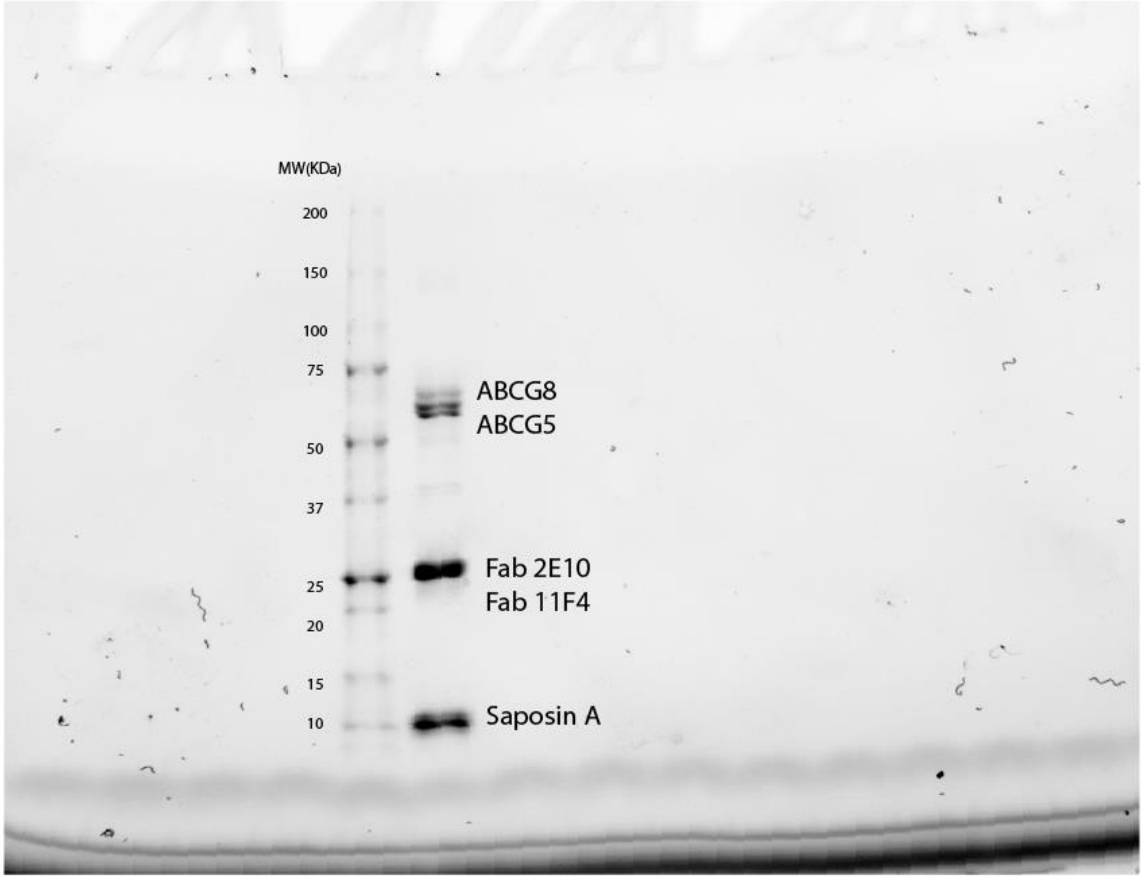

Supplementary figure 1. SDS-PAGE of ABCG5/G8 in complex with Fab 2E10 and Fab 11F4 reconstituted in Saposin A nanodiscs.

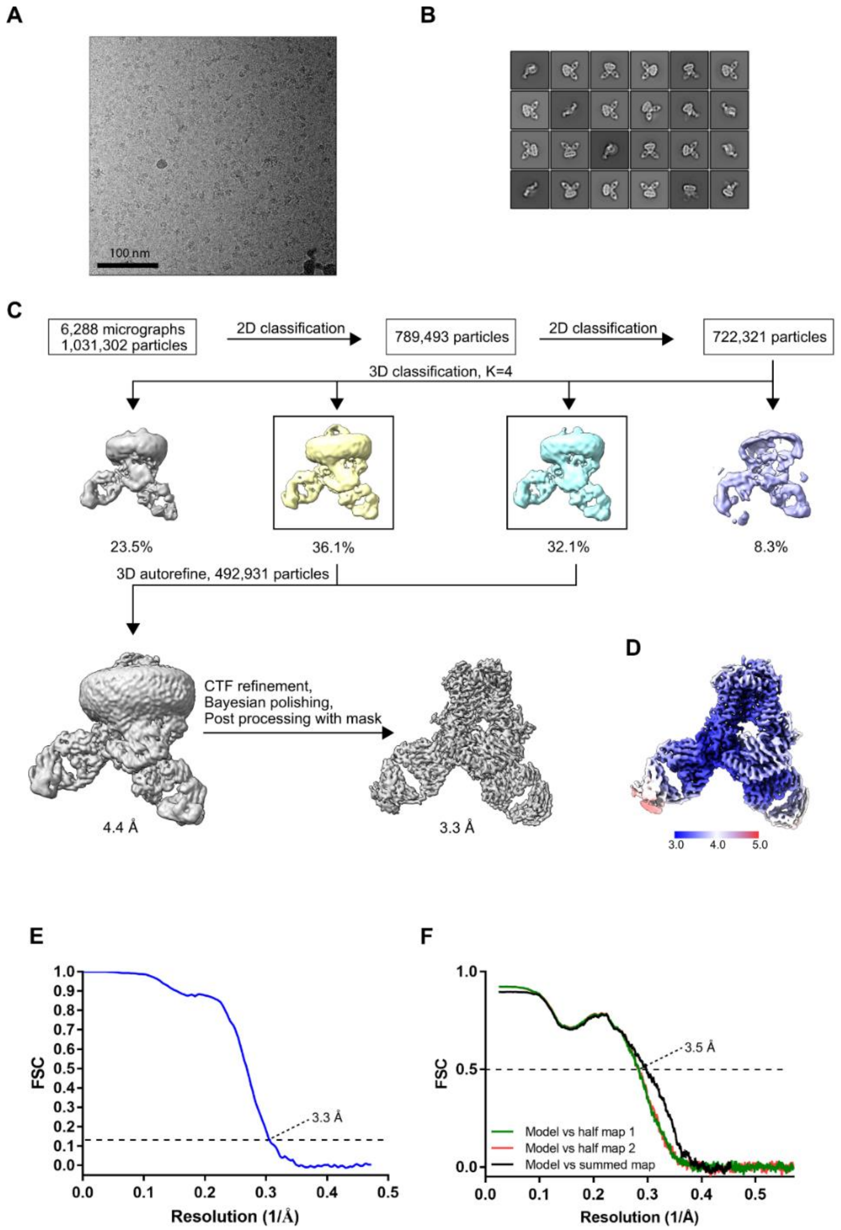

Supplementary figure 2. Cryo-EM analysis of the ABCG5/G8-Fab complex. (A) Representative cryo-EM micrograph and (B) 2D class averages. (C) Flow chart of data processing. Details can be found in the Image processing section. (D) Local resolution estimated by ResMap. (E) The gold-standard FSC curve for the cryo-EM map. (F) FSC curves for the refined model versus the summed 3.5 Å map (black curve), the refined model versus half map 1 (green curve) and the refined model versus half map 2 (red curve).

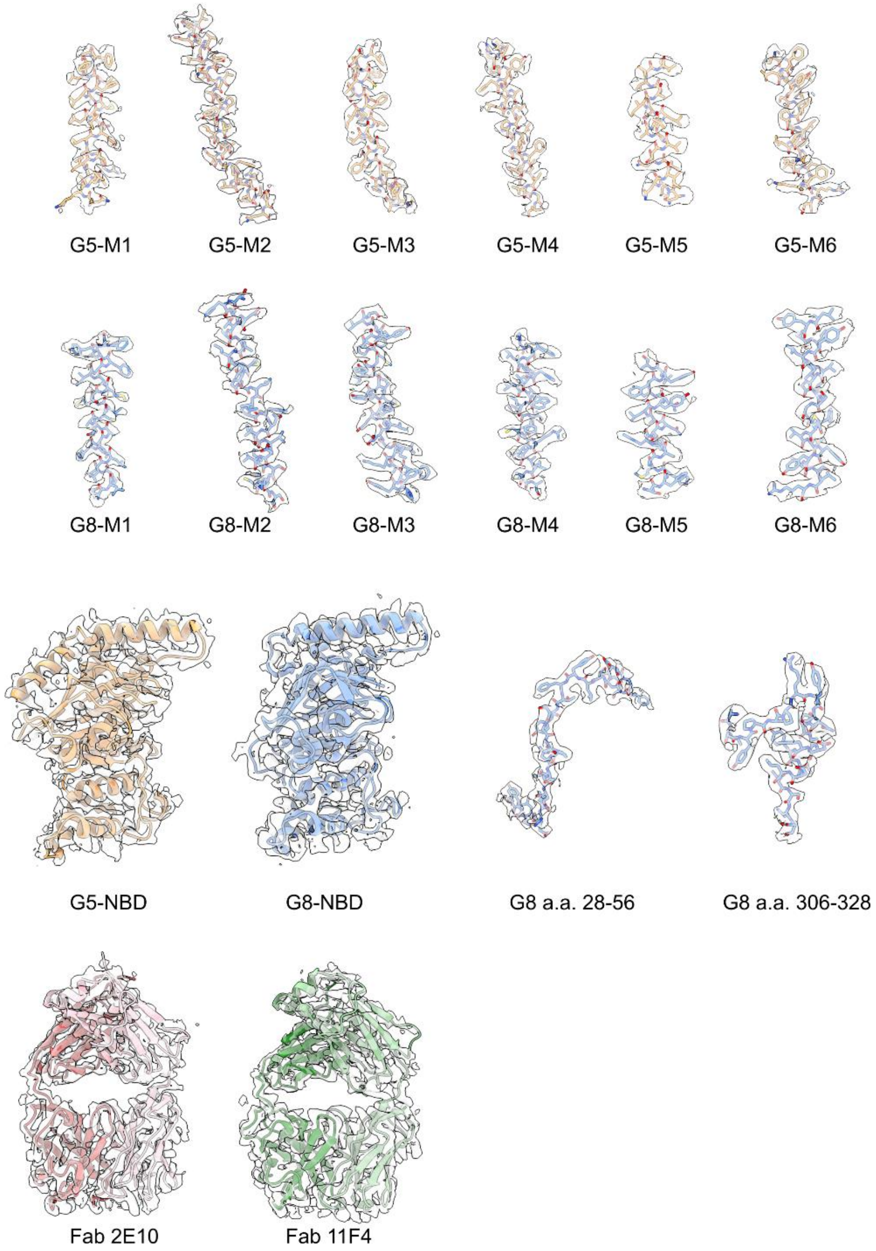

Supplementary figure 3. EM map of the ABCG5/G8-Fab complex at the 5σ level. Representative regions of the EM map of the ABCG5, ABCG8, Fab 2E10 and Fab 11F4.

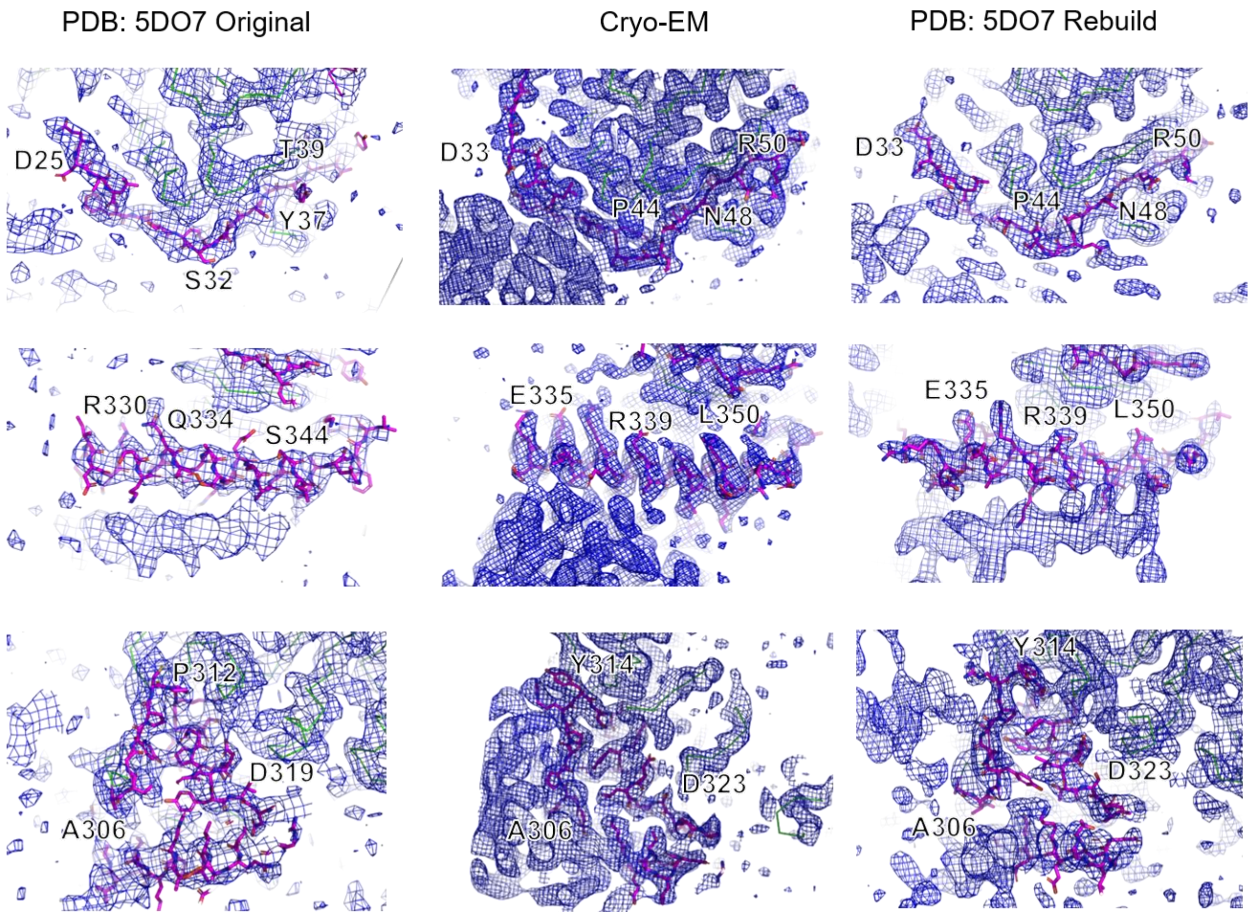

Supplementary figure 4. Reevaluation of ABCG5/G8 crystal structure (PDB: 5DO7). Left and middle: electron density maps of ABCG5/G8 crystal structure (left, PDB: 5DO7, 2F0-Fc) and cryo-EM structure (middle, structural factor) are shown in blue meshes contoured at 1.0 σ. The residues mismatched in the structures are shown in magenta sticks. Right: electron density map (2F0-Fc) of 5DO7 rebuilt and refined based on the cryo-EM model (R_{free}: 0.324, R_{work}: 0.265). The rebuilt residues are shown in magenta sticks.

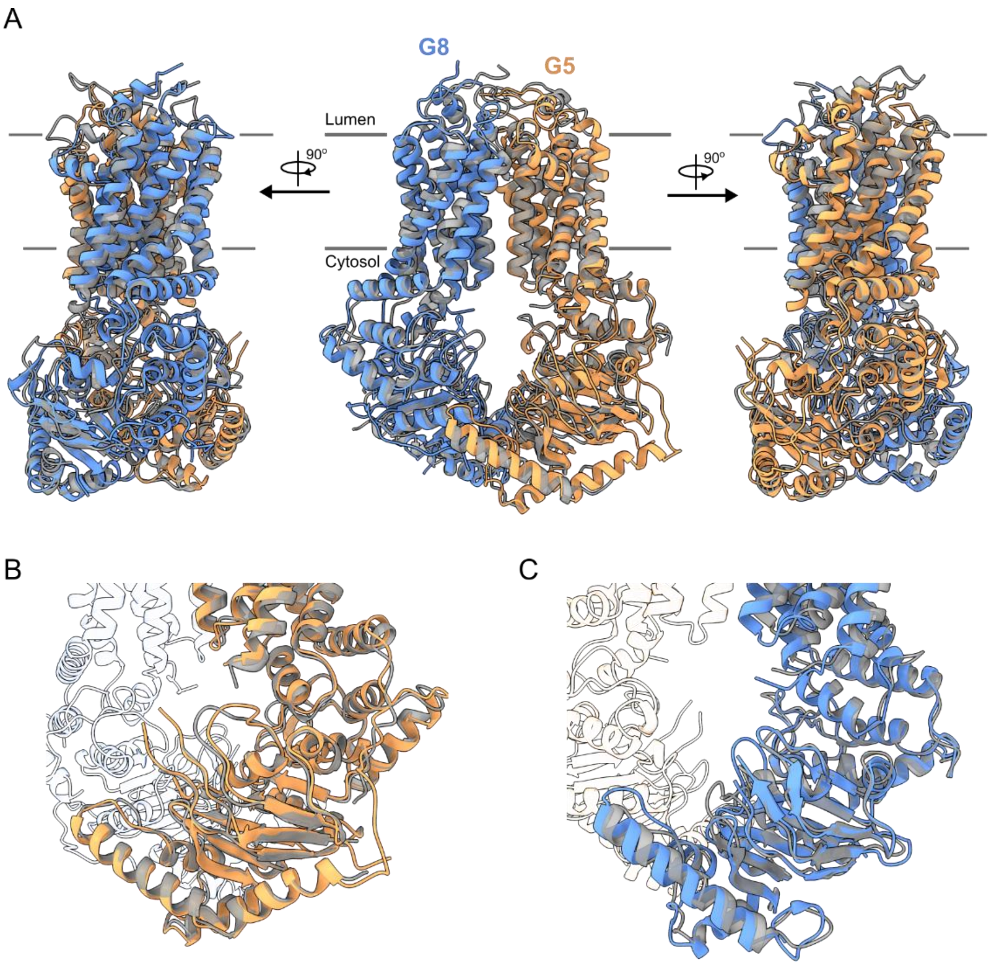

Supplementary figure 5. Structural comparison between the cryo-EM structure and crystal structure of ABCG5/G8. (A) Overall comparison between the cryo-EM structure (orange: ABCG5, blue: ABCG8) and crystal structure (gray) of ABCG5/G8. (B) Structural comparison between the NBD domain of ABCG5 from the cryo-EM structure (orange) and crystal structure (gray). (C) Structural comparison between the NBD domain of ABCG8 from the cryo-EM structure (blue) and crystal structure (gray).

```
Human ABCG1    247 LDSASCFQVVSLMKGLAQGGRSIICTIHQPSAKLFELFDQLYVLSQGQCVYRGKVCNLVPYLRDLGLNCP 316
Human ABCG2    216 LDSSTANAVLLLLKRMSKQGRTIIFSIHQPRYSIFKLFDSLTLLASGRLMFHGPAQEALGYFESAGYHCE 285
Human ABCG4    231 LDSASCFQVVSLMKSLAQGGRTIICTIHQPSAKLFEMFDKLYILSQGQCIFKGVVTNLIPYLKGLGLHCP 300
Human ABCG5    223 LDCMTANQIVVLLVELARRNRIVVLTIHQPRSELFQLFDKIAILSFGELIFCGTPAEMLDFFNDCGYPCP 292
Human ABCG8    243 LDSFTAHNLVKTLSRLAKGNRLVLISLHQPRSDIFRLFDLVLLMTSGTPIYLGAAQHMVQYFTAIGYPCP 312
Mouse ABCG5    224 LDCMTANQIVLLLAELARRDRIVIVTIHQPRSELFQHFDKIAILTYGELVFCGTPEEMLGFFNNCGYPCP 293
Mouse ABCG8    244 LDSFTAHNLVTTLSRLAKGNRLVLISLHQPRSDIFRLFDLVLLMTSGTPIYLGAAQQMVQYFTSIGHPCP 313

Human ABCG1    317 TYHNPADFVMEVASGEYGDQNSR-LVRAVREGMCDSDHKRD---LGGDA--EVNPFLWHRPSEEVKQTKR 380
Human ABCG2    286 AYNNPADFFLDIINGDSTAVALNREEDFKATEIIEPSKQDK-PLIEKLAEIYVNSSFYKETKAELHQLSG 354
Human ABCG4    301 TYHNPADFIIEVASGEYGDLNPM-LFRAVQNGLCAMAEKKS---SPEKN--EVPAPCPPCP-PEVD---- 359
Human ABCG5    293 EHSNPFDFYMDLTSVDTQSKERE-IETSKRVQMIESAYKKS---A-----------ICHKTLKNIERMKH 347
Human ABCG8    313 RYSNPADFYVDLTSIDRRSR-------EQELATREKAQSLAALFLEKVR--DLDDFLWKAETKDLDEDTC 373
Mouse ABCG5    294 EHSNPFDFYMDLTSVDTQSRERE-IETYKRVQMLECAFKES---D-----------IYHKILENIERARY 348
Mouse ABCG8    314 RYSNPADFYVDLTSIDRRSK-------EREVATVEKAQSLAALFLEKVQ--GFDDFLWKAEAKELNTST- 373
                      NPXDFXXD
```

Supplementary figure 6. Sequence alignments of amino acid residues across NPXDFXXD motif. The conserved lysine/arginine and aspartate/glutamate for NBD dimerization in the ABCG family proteins are highlighted.

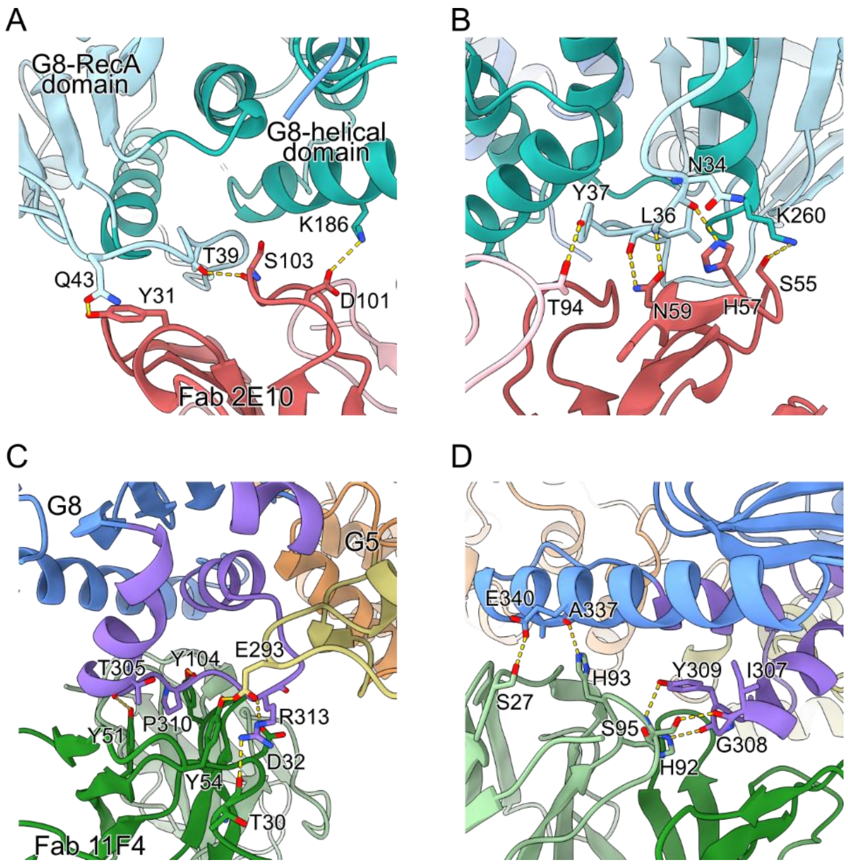

Supplementary figure 7. Detail analysis of the interaction between Fab 2E10 and ABCG8 and between Fab 11F4 and ABCG5/G8. (A) (B) Hydrogen bonds and charge interactions between Fab 2E10 and ABCG8 are shown as dashed lines. Heavy chain and light chain of Fab 2E10 are colored in red and light red, respectively. (C) (D) Hydrogen bonds and charge interactions between Fab 11F4 and ABCG5/G8 are shown as dashed lines. The three-helix bundle with NPXDFXXD motif of ABCG5 and ABCG8 are colored in yellow and purple, respectively. Heavy chain and light chain of Fab 11F4 are colored in green and light green, respectively.

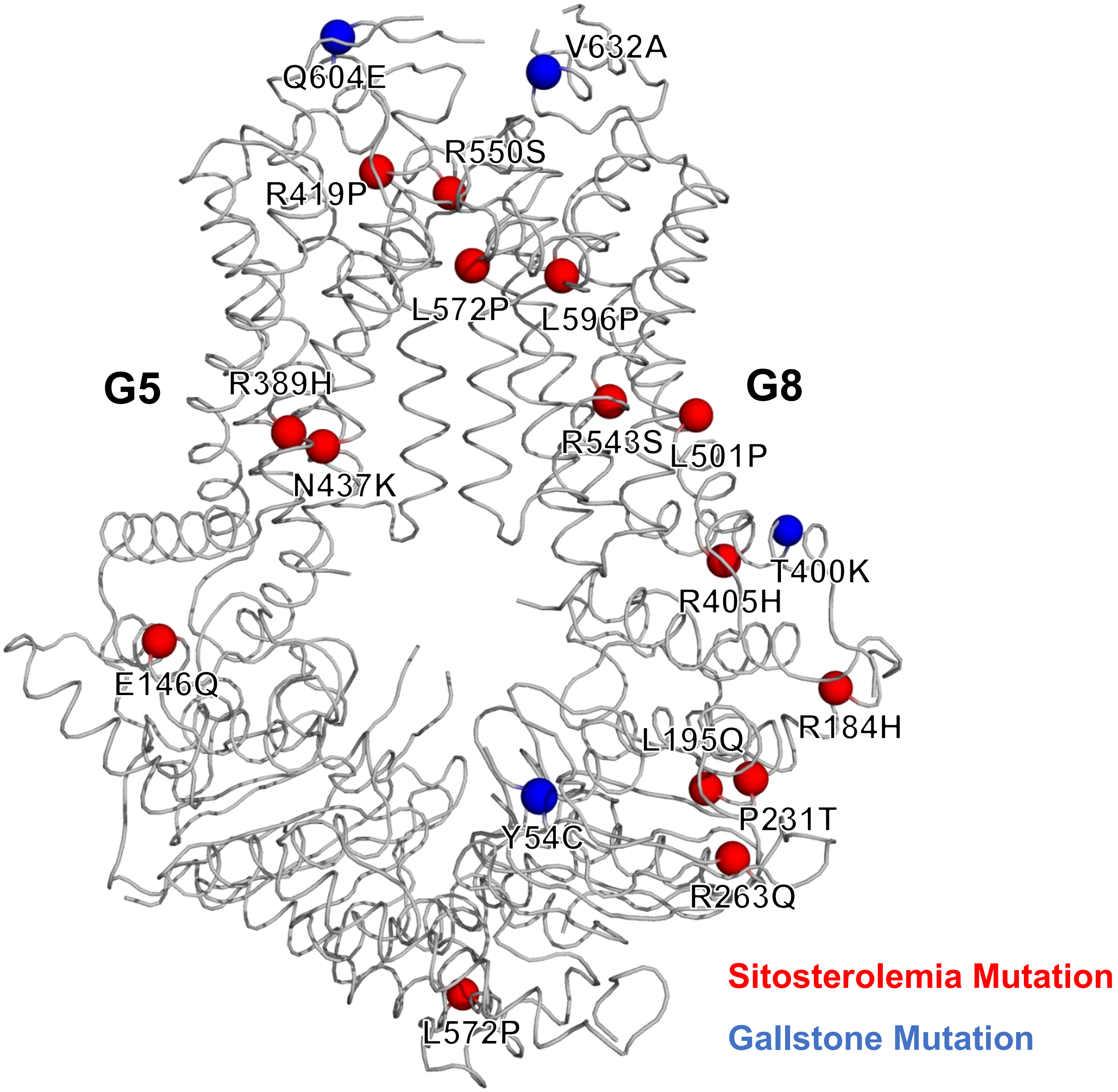

Supplementary figure 8. Disease related missense mutations in ABCG5/G8. Cartoon representation of ABCG5/G8 viewed within the plane of the membrane. Sitosterolemia related mutations are labeled in red sphere; Gallstone related mutations are labeled in blue sphere. The mutations are adapted from a review paper published by Zein *et al*[16].
