# Supplementary material for: Cryo-EM structure of ABCG5/G8 in complex with modulating antibodies
Source: Commun Biol. 2021 May 5;4:526. doi: 10.1038/s42003-021-02039-8 (PMC8100176; doi:10.1038/s42003-021-02039-8)
Supplement: Supplementary file 3 — Description of Additional Supplementary Files [file 42003_2021_2039_MOESM3_ESM.pdf]

Description of Additional Supplementary Files

File name: Supplementary Data 1

Description: Raw data underlying Figs. 1a, b, c, d, and 3a
